# A comparative study of early postoperative pain: robotic-assisted versus conventional total knee arthroplasty

**DOI:** 10.1007/s00264-025-06451-1

**Published:** 2025-03-05

**Authors:** Keerati Chareancholvanich, Chaturong Pornrattanamaneewong, Ronnakit Udompanich, Kit Awirotananon, Rapeepat Narkbunnam

**Affiliations:** Department of Orthopaedic Surgery, Faculty of Medicine Siriraj Hospital, Mahidol University, Bangkok, 10700, Thailand

**Keywords:** TKA, Robotic-assisted, Total, Knee, Arthroplasty

## Abstract

**Purpose:**

While robotic-assisted total knee arthroplasty (RA-TKA) has demonstrated improved surgical precision, its impact on early postoperative pain management remains unclear. This study compared early postoperative pain outcomes between RA-TKA and conventional TKA (C-TKA).

**Methods:**

In this retrospective study, 230 consecutive patients (309 knees) who underwent primary TKA were analyzed: 143 patients (181 knees) in the C-TKA group and 87 patients (128 knees) in the RA-TKA group. Pain scores at rest and during movement were assessed using the Numerical Pain Rating Scale for 72 h postoperatively. Secondary outcomes included opioid consumption and length of hospital stay.

**Results:**

While pain scores at rest showed no significant differences between groups, RA-TKA patients reported significantly lower pain scores during movement at 24 h post-surgery (*p* = 0.023). The RA-TKA group demonstrated significantly reduced opioid consumption during the first 48 postoperative hours (*p* = 0.001 for 0-24 h; *p* = 0.03 for 24-48 h) and shorter length of hospital stay (*p* = 0.011). Subgroup analysis of unilateral procedures showed similar advantages in the RA-TKA group.

**Conclusion:**

RA-TKA was associated with reduced pain during movement, decreased opioid consumption, and shorter hospital stay in the early postoperative period compared to C-TKA.

## Background

Total knee arthroplasty (TKA) is a well-established surgical procedure for treating advanced knee osteoarthritis(OA), providing significant pain relief and functional improvement for patients [1]. However, managing postoperative pain remains a critical challenge in TKA, as it directly impacts patient satisfaction, early mobilization, and overall recovery [2]. In recent years, robotic-assisted TKA has emerged as a promising technology aimed at improving surgical precision and potentially enhancing patient outcomes [3, 4].

Robotic-assisted TKA(RA-TKA) systems, such as the MAKO system (Stryker) [5], offer several potential advantages over conventional TKA(C-TKA) techniques. These include enhanced preoperative planning through 3D modeling, improved intraoperative precision in bone cuts and implant positioning, and the potential for better soft tissue balancing [6, 7]. Moreover, there are notable angular differences between the mechanical axes referenced by robotic systems and the anatomical axes used in conventional TKA, which may affect surgical outcomes and postoperative evaluation [8]. While studies have shown that robotic-assisted TKA can lead to more accurate component positioning and improved mechanical alignment [9], evidence regarding its influence on acute postoperative pain intensity and early functional recovery remains inconclusive [10–15].

Pain management in the immediate postoperative period is crucial for TKA patients, as it influences early mobilization, rehabilitation progress, and length of hospital stay [16]. Effective pain control can also reduce the need for opioid medications, which is particularly important given the current emphasis on minimizing opioid use in postoperative care [17]. However, comparative data on early postoperative pain outcomes between robotic-assisted and conventional TKA are limited, particularly in studies with large sample sizes and frequent pain assessments. The present study aims to address this gap in knowledge by conducting a comprehensive comparison of early postoperative pain outcomes between robotic-assisted and conventional TKA.

## Methods

### Study design and participants

This retrospective study, approved by our Institutional Review Board, examined 230 consecutive patients who underwent primary TKA at our institute between January 2021 and July 2023. The cohort comprised 143 patients (181 knees) who underwent C-TKA, followed by 87 patients (128 knees) who underwent RA-TKA. All procedures were performed by the same surgical team of three arthroplasty surgeons (K.C., R.N., C.P.), who were highly experienced in conventional and robotic-assisted TKA techniques. Inclusion criteria were patients with advanced symptomatic knee OA (Kellgren–Lawrence grade ≥ 3) and surgical indication for TKA. Patients were excluded if they had previous high tibial or distal femoral osteotomy, post-traumatic OA, revision of femoral or tibial components, or experienced postoperative complications.

### Surgical procedure

Preoperative planning for C-TKA involved standing full-length extremity X-rays when femoral or tibial deformities were suspected, while RA-TKA patients underwent full-length extremity computed tomography (CT) scans using the MAKO system (Stryker) protocol. This protocol enabled personalized planning and 3D knee modeling, establishing implant size and position based on the mechanical axis in multiple planes.

All patients were positioned supine, with a pneumatic tourniquet applied and inflated after draping. C-TKA procedures used various implants (Persona; Zimmer, Smith & Nephew; Anthem, or Triathlon; Stryker) based on surgeon preference, while all RA-TKA utilized the MAKO system with Triathlon; Stryker prosthesis. The patella was not resurfaced in any procedure. All surgeries employed a midline incision with a standard medial parapatellar approach.

C-TKAs were performed using a measured resection technique with standard instrumentation, aiming for neutral alignment. Intramedullary guides were used for femoral cuts and extramedullary guides for tibial cuts. Sequential bone cuts were made on the distal femur, proximal tibia, and condyles, followed by ligament releases as needed.

For RA-TKA, the procedure began with placement of registration arrays and creation of a 3D model for intraoperative navigation. Dynamic navigation allowed for adjustment of component positioning and alignment. Bone cuts were made using the robotic arm with haptic feedback, followed by manual soft tissue management.

In both techniques, alignment and gaps were verified, and the articular capsule received local anaesthesia. For all surgeries, definitive implants were cemented with fixed bearings, followed by similar fashion of tissue closure without drains.

### Anesthesia and post-operative management

The anaesthetic protocol was standardized for both groups, consisting of regional anesthesia with adductor canal blocks administered by highly experienced anesthesiologists specializing in orthopaedics. Post-operatively, all patients received a tailored analgesic protocol including acetaminophen, NSAIDs, and gabapentin, with intravenous morphine (1–2 mg) used as rescue medication if pain exceeded 3 on the Numerical Pain Rating Scale. The rehabilitation program, initiated on the day of surgery, included intermittent pneumatic compression devices and ankle pumping exercises to prevent deep vein thrombosis, passive and active knee motion exercises, and full weight-bearing with a walker. Patients were discharged when they achieved adequate pain control with oral analgesia, knee flexion of at least 90 degrees in both active and passive motion, and independent ambulation with a walker, including the ability to navigate stairs.

### Outcomes assessment

All demographic data and patient outcomes were retrospectively collected by one independent assessor from electronic medical records. Baseline measurements included: age at time of surgery (years), gender (male/female), body mass index (kg/m²), race, ASA grade, side of intervention (right/left) and pre-operative range of motion (ROM) (degrees).

Pain assessment was a key focus of the study, aimed at evaluating differences between the two groups during the early postoperative period. Numerical Pain Rating Scale (NPRS) scores were diligently recorded by independent nursing staff for up to 72 h postoperatively. Resting pain was assessed at four hour intervals for the first 72 h following surgery. Pain during activity was evaluated once daily after physical therapy sessions, which encompassed gait training and knee flexion exercises. Independent physical therapists recorded these activity-related pain measurements.

Other outcomes collected by the independent assessor included morphine consumption (milligrams), length of hospital stay (days), and postoperative range of motion (degrees).

### Statistical analysis

SPSS 18.0 (SPSS Inc., Chicago, IL, USA) software was used for statistical analysis. Continuous measurements are expressed as mean and range. When the parametric test assumptions were met after the examination of distributions of all variables, the student *t*-test was used in independent groups and Mann–Whitney U-test was used if there was no difference between the groups in terms of numerical variables. *χ*2 test was used for categorical variables. Two-way mixed repeated ANOVA was performed for each timepoint. The α level was set at *P* < 0.05 for two-tailed comparisons.

## Results

There were no significant differences in baseline characteristics between C-TKA and RA-TKA groups. The demographics of patients are shown in Table 1. Pain scores at rest (NPRS) showed no significant differences between the groups across all time intervals (0–24, 24–48, and 48–72 h). However, patients who underwent C-TKA exhibited significantly higher pain scores during movement at 24 h post-surgery (*p* = 0.023) compared to the RA-TKA group (Table 2). Opioid consumption was significantly higher in the C-TKA group during the first 24 h (*p* = 0.001) and 24–48 h (*p* = 0.03) post-surgery (Table 3). A subgroup analysis of unilateral TKA patients revealed decreased pain scores on movement at 72 h (*p* = 0.04) and reduced opioid consumption in the 0–24 and 24–48 h periods (*p* = 0.03 and *p* = 0.02, respectively) for the robotic-assisted group (Table 4). Other outcomes showed a significantly longer length of stay (LOS) for patients in the conventional TKA group (*p* = 0.011). However, no statistically significant differences were observed between the groups in postoperative range of motion (ROM) on days 1, 2, and 3 (Table 3).

**Table 1:** Demographic data of all treatment groups

Demographic Data	Conventional TKA (*n* = 143)	Robotic-assisted TKA (*n* = 87)	*p*-value
Gender Female Male	110 (76.9%) 33 (23.1%)	58 (66.7%) 29 (33.3%)	0.094
Age (years)	70.01 ± 7.46	68.57 ± 7.89	0.257^a^
Body weight (kilograms)	65.31 ± 11.78	67.76 ± 13.15	0.145
Body height (cm)	155.45 ± 8.73	157.05 ± 8.99	0.194^a^
BMI(kg/m^2^)	27.08 ± 4.70	27.39 ± 4.27	0.618
Side of operation Right Left Bilateral	58 (40.6%) 47 (32.9%) 38 (26.6%)	20 (23.0%) 26 (29.9%) 41 (47.1%)	0.006
Pre-op ROM (degree) Flexion Extension	103.91 ± 26.07 3.94 ± 5.48	105.46 ± 19.90 4.92 ± 5.77	0.545^a^ 0.165^a^

a: Mann-Whitney U test

**Table 2:** The primary outcome comparison conventional and robotic-assisted TKA

Outcome	Conventional TKA	Robotic-assisted TKA	*p*-value
**NPRS at rest**			
At 0–24 h	0.96 ± 0.93 0.71 (0.14–1.43)	0.82 ± 0.94 0.57 (0–1.14)	0.170^a^
At 28–48 h	0.69 ± 0.88 0.33 (0–1.17)	0.53 ± 0.69 0.33 (0–0.83)	0.565^a^
At 52–72 h	0.39 ± 0.64 0 (0–0.67)	0.26 ± 0.43 0 (0–0.33)	0.348^a^
**NPRS at movement**			
At 24 h	3.76 ± 2.65 4 (2–5)	2.94 ± 2.51 3 (0–5)	*0.023* ^a^
At 48 h	2.85 ± 2.47 3 (0–4)	2.17 ± 1.86 2 (0–3)	0.077^a^
At 72 h	1.90 ± 1.99 2 (0–3)	1.46 ± 1.74 1 (0–3)	0.148^a^

a: Mann-Whitney U test

**Table 3:** The secondary outcomes comparison conventional and robotic-assisted TKA

Outcome	Conventional TKA	Robotic-assisted TKA	*p*-value
Length of stay (days)	4.15 ± 1.31 4 (3–5)	4.71 ± 2.16 4 (4–5 )	*0.011* ^a^
Opioid Consumption 0–24 h	1.94 ± 2.53 1 (0–2.5)	0.99 ± 1.39 0 (0–2)	*0.001* ^a^
Opioid Consumption 24–48 h	1.28 ± 2.30 0 (0–2)	0.68 ± 1.22 0 (0–1)	*0.030* ^a^
Opioid Consumption 48–72 h	0.49 ± 1.55 0 (0–0)	0.33 ± 0.67 0 (0–0)	0.968^a^
**ROM: Flexion (degree)**			
Post-op Day1	74.02 ± 22.30 90 (60–90)	68.05 ± 29.86 90 (60–90)	0.413^a^
Post-op Day2	79.15 ± 20.94 90 (80–90)	74.75 ± 29.21 90 (85–90)	0.885^a^
Post-op Day3	82.93 ± 19.86 90 (90–90)	80.26 ± 25.05 90 (90–90)	0.715^a^
**ROM: Extension (degree)**			
Post-op Day1	4.36 ± 6.71 0 (0–10)	3.79 ± 6.02 0 (0–10)	0.571^a^
Post-op Day2	2.64 ± 4.42 0 (0–10)	2.81 ± 4.70 0 (0–10)	0.828^a^
Post-op Day3	2.29 ± 4.19 0 (0–0)	2.76 ± 4.58 0 (0–10)	0.372^a^

a: Mann-Whitney U test

**Table 4:** The comparison of outcomes in unilateral conventional and robotic-assisted TKA

Outcome	Conventional TKA	Robotic-assisted TKA	*p*-value
**NPRS at movement**			
At 24 h	3.61 ± 2.62 4 (2.5–5)	2.74 ± 2.35 3 (0–5)	0.074^a^
At 48 h	2.72 ± 2.34 3 (2–4)	2.04 ± 1.90 2 (0.5–3.5)	0.100^a^
At 72 h	1.78 ± 1.87 2 (0–3)	1.07 ± 1.34 1 (0–2)	*0.044* ^a^
**Opioid Consumption**			
At 0–24 h	2.02 ± 2.70 1 (0–2.5)	0.86 ± 1.30 0 (0–1.63)	*0.003* ^a^
At 24–48 h	1.43 ± 2.57 0 (0–2)	0.54 ± 1.00 0 (0–1)	*0.021* ^a^
At 48–72 h	0.53 ± 1.71 0 (0–0)	0.27 ± 0.61 0 (0–0)	0.633^a^

a: Mann-Whitney U test

## Discussion

This study, comparing early postoperative outcomes between RA-TKA and C-TKA, provides valuable insights into robotic technology’s benefits in orthopaedic surgery. Our large sample size (230 patients, 309 knees) provides robust statistical power. Our findings offer a comprehensive understanding of pain, opioid consumption, and early recovery postoperatively. Patients who underwent RA-TKA experienced significantly lower pain scores during movement at 24 h post-surgery compared to C-TKA patients. This aligns with previous studies reporting improved pain outcomes with RA-TKA. Kayani et al. [6] compared 40 C-TKA and 40 MAKO RA-TKA patients, concluding that RA-TKA led to decreased pain, improved early functional recovery, and reduced hospital stay. Batailler et al.‘s [15] systematic review of 26 MAKO RA-TKA studies showed improved postoperative pain, prosthetic positioning, and functional outcomes compared to C-TKA. These benefits may be attributed to the MAKO robot’s precise bone cutting, enabled by preoperative planning software, real-time intraoperative feedback, and haptically bounded saw blade. These features help protect surrounding soft tissues and ligaments [18, 19]. Investigating the mechanisms, a study showed MAKO RATKA resulted in a milder early postoperative systemic inflammatory response compared to CTKA [20]. Our study found significant differences in pain scores during movement between RA-TKA and C-TKA groups, but not at rest. This distinction is crucial, as pain during motion impacts early rehabilitation and functional outcomes. The reduced pain during movement in RA-TKA patients may enhance early rehabilitation, leading to improved functional outcomes and higher patient satisfaction, as reported in previous studies [21].

Reduced opioid consumption in the RA-TKA group during the first 48 h post-surgery is noteworthy. This suggests RA-TKA may contribute to more effective pain management, potentially reducing opioid-related complications and dependency [14, 22]. Given the ongoing opioid epidemic, surgical techniques reducing opioid use without compromising pain control are of significant clinical interest. The shorter length of stay (LOS) in the RA-TKA group has significant implications for healthcare systems and patient satisfaction. Reduced LOS is associated with cost savings and improved patient outcomes [23]. RA-TKA’s benefits may extend beyond postoperative pain management to influence patient recovery and healthcare resource utilization. This has implications for hospital efficiency, patient throughput, and overall healthcare costs.

Subgroup analysis of unilateral TKA patients revealed decreased pain scores on movement at 72 h and reduced opioid consumption in the first 48 h for the RA-TKA group. These benefits may be particularly pronounced in unilateral procedures, possibly due to less overall injury compared to bilateral procedures. The non-operated leg may help compensate for patient movement in the early postoperative period. These findings are consistent with previous studies [24–26].

It is important to note that several recent meta-analyses have reported comparable outcomes between RA-TKA and C-TKA across multiple parameters, including pain scores, clinical outcomes, radiological outcomes, and patient satisfaction [12, 27–29]. These findings warrant careful interpretation, as they aggregate data from studies utilizing diverse robotic platforms and varying knee alignment philosophies that have evolved over time. The heterogeneity in robotic assistance technologies suggests that individual robotic systems may offer distinct advantages that could be obscured in pooled analyses. As highlighted by Vermue et al., each robotic system’s unique features and workflows necessitate independent evaluation to accurately determine their specific contributions to surgical outcomes [30].

Our study has several limitations to consider. The retrospective design introduces potential selection bias, despite our efforts to mitigate this through strict inclusion criteria. While the RA-TKA group consistently used the MAKO system, the C-TKA group employed various prosthesis designs, which could potentially influence outcomes and introduce confounding variables. As a single-centre study with experienced surgeons, the generalizability of our findings may be limited. Given our study’s focus on immediate postoperative outcomes within the first 72 h, when patients are primarily concerned with pain management and basic mobility, comprehensive functional outcomes and patient-reported measures were not feasible during this acute recovery phase. This early postoperative timeframe naturally limits our ability to assess broader aspects of patient satisfaction and functional recovery.

## Conclusion

This comparative study demonstrates that robotic-assisted TKA offers several significant benefits over conventional TKA in the early postoperative period. Patients who underwent robotic-assisted TKA experienced less pain during movement at 24 h post-surgery, required reduced opioid consumption in the first 48 h, and had a significantly shorter length of hospital stay. These findings suggest potential advantages of robotic assistance in the acute post-operative phase of total knee arthroplasty.

## Data Availability

No datasets were generated or analysed during the current study.
